# *Chlamydia trachomatis* Is Resistant to Inclusion Ubiquitination and Associated Host Defense in Gamma Interferon-Primed Human Epithelial Cells

**DOI:** 10.1128/mBio.01417-16

**Published:** 2016-12-13

**Authors:** Arun K. Haldar, Anthony S. Piro, Ryan Finethy, Scott T. Espenschied, Hannah E. Brown, Amanda M. Giebel, Eva-Maria Frickel, David E. Nelson, Jörn Coers

**Affiliations:** a Departments of Molecular Genetics and Microbiology and Immunology, Duke University Medical Center, Durham, North Carolina, USA; b Department of Microbiology and Immunology, Indiana University School of Medicine, Indianapolis, Indiana, USA; c The Francis Crick Institute, Host-Toxoplasma Interaction Laboratory, London, United Kingdom

## Abstract

The cytokine gamma interferon (IFN-γ) induces cell-autonomous immunity to combat infections with intracellular pathogens, such as the bacterium *Chlamydia trachomatis*. The present study demonstrates that IFN-γ-primed human cells ubiquitinate and eliminate intracellular *Chlamydia*-containing vacuoles, so-called inclusions. We previously described how IFN-γ-inducible immunity-related GTPases (IRGs) employ ubiquitin systems to mark inclusions for destruction in mouse cells and, furthermore, showed that the rodent pathogen *Chlamydia muridarum* blocks ubiquitination of its inclusions by interfering with mouse IRG function. Here, we report that ubiquitination of inclusions in human cells is independent of IRG and thus distinct from the murine pathway. We show that *C. muridarum* is susceptible to inclusion ubiquitination in human cells, while the closely related human pathogen *C. trachomatis* is resistant. *C. muridarum*, but not *C. trachomatis*, inclusions attract several markers of cell-autonomous immunity, including the ubiquitin-binding protein p62, the ubiquitin-like protein LC3, and guanylate-binding protein 1. Consequently, we find that IFN-γ priming of human epithelial cells triggers the elimination of *C. muridarum*, but not *C. trachomatis*, inclusions. This newly described defense pathway is independent of indole-2,3-dioxygenase, a known IFN-γ-inducible anti-*Chlamydia* resistance factor. Collectively, our observations indicate that *C. trachomatis* evolved mechanisms to avoid a human-specific, ubiquitin-mediated response as part of its unique adaptation to its human host.

## IMPORTANCE

*Chlamydia trachomatis* is the leading cause of sexually transmitted bacterial infections and responsible for significant morbidity, including pelvic inflammatory disease, infertility, and ectopic pregnancies in women. As an obligate intracellular pathogen, *C. trachomatis* is in perpetual conflict with cell-intrinsic defense programs executed by its human host. Our study defines a novel anti-*Chlamydia* host resistance pathway active in human epithelial cells. This defense program promotes the deposition of the small antimicrobial protein ubiquitin on vacuoles containing *Chlamydia*. We show that this ubiquitin-based resistance pathway of human cells is highly effective against a *Chlamydia* species adapted to rodents but ineffective against human-adapted *C. trachomatis*. This observation indicates that *C. trachomatis* evolved strategies to avoid entrapment within ubiquitin-labeled vacuoles as part of its adaptation to the human innate immune system.

## INTRODUCTION

The intracellular bacterial pathogen *Chlamydia trachomatis* is among the most common causative agents of sexually transmitted infections. According to the World Health Organization, an estimated 100 million individuals are infected per annum (1). Many of these infections lead to disease and irreparable pathologies; *C. trachomatis* infections frequently result in urethritis in men and pelvic inflammatory disease, tubal factor infertility, and ectopic pregnancies in women (2–4). *C. trachomatis*-associated diseases in women are due to extensive pathogen exposure stemming from chronic, recurring, or repeat infections, all indicative of an inability of the human immune system to promptly sterilize *C. trachomatis* infections or to establish effective immune memory. The failure of our immune system to protect against *C. trachomatis* infections is likely the consequence of active or passive immune evasion by this stealth pathogen (2–8).

*C. trachomatis* is an obligate intracellular pathogen that resides and replicates within the confines of specialized intracellular vacuoles termed “inclusions” (9). *C. trachomatis* establishes an infection by taking primary residency inside epithelial cells. *C. trachomatis* enters epithelial cells in its infectious form known as the elementary body (EB) and then differentiates into the replicative reticulate body (RB). Following several rounds of binary fission within the inclusion, RBs begin to differentiate back into EBs, which then exit the spent host cell (9, 10). While naive epithelial cells are permissive for intracellular *C. trachomatis* growth, priming of human cells with the proinflammatory cytokine gamma interferon (IFN-γ) inhibits the ability of *C. trachomatis* to complete its developmental cycle (11).

IFN-γ is predominantly produced by lymphocytes in response to an infection, yet its cognate receptor is expressed in virtually all cell types (12). Priming of cells with IFN-γ induces the expression of hundreds of IFN-stimulated genes (ISGs), which control an extensive network of cell-autonomous defense programs (8, 12, 13). In human epithelial cells, IFN-γ-activated cell-autonomous immunity to *C. trachomatis* is mediated by the enzyme indole-2,3-dioxygenase (IDO). IDO metabolizes host cell tryptophan and thereby depletes intracellular tryptophan stores. Because *C. trachomatis* is a tryptophan auxotroph, tryptophan depletion restricts intracellular replication of *C. trachomatis* (14–16). In response to tryptophan starvation, *C. trachomatis* scavenges extracellular indole from its surrounding microbial community and thereby counteracts IDO-mediated nutritional immunity (6, 8, 17, 18). However, it has remained unknown whether and how *C. trachomatis* resists immunity executed by any human ISGs other than IDO.

In mice, the human-restricted pathogen *C. trachomatis* is quickly eliminated through IFN-γ-mediated immune responses that are independent of IDO (19–22). A forward genetic screen approach identified IFN-γ-inducible immunity-related GTPases (IRGs) as critical host resistance factors that execute sterilizing immunity against *C. trachomatis* in mice (20, 23). Members of the IRG protein family function cooperatively to detect the locations of inclusions within host cells (24). Following binding to inclusions, IRG proteins recruit E3 ligases, such as tumor necrosis factor receptor-associated factor 6 (TRAF6) and tripartite motif-containing protein 21 (TRIM21) and thereby promote the deposition of ubiquitin on unknown substrates associated with inclusion membranes (25). Ubiquitinated *C. trachomatis* inclusions become targets for the ubiquitin-binding protein p62, which escorts antimicrobial guanylate-binding proteins (GBPs) to inclusions. The IRG-dependent ubiquitination of inclusions ultimately results in inclusion rupture, the release of bacteria into the host cell cytosol (25), and the engulfment of the ejected bacteria inside degradative autolysosomes (26).

Mouse IRG proteins can be placed into two subgroups that are defined by the amino acid sequence of their GTP binding pockets and by their subcellular localization. The majority of IRG proteins feature a canonical GXXXXGKS sequence in the P-loop of the GTP binding site and are accordingly referred to as “GKS” proteins (24, 27). GKS proteins are predominantly found in the host cell cytosol yet are able to translocate to inclusion membranes upon infection with *C. trachomatis* (28). This inclusion targeting event is regulated by the second subgroup of the IRG protein family, the IRG family M (IRGM) proteins (29), which are defined by their noncanonical GXXXXGMS P-loop sequence (27). While GKS proteins bind to inclusions, IRGM proteins instead associate with host cell organelles and prevent GKS binding to these self-structures (24, 29). Membrane-bound IRGM proteins undergo transient interactions with GKS proteins and maintain GKS proteins in their inactive, monomeric GDP-bound state (30). GKS monomers acquire GTP, oligomerize, and bind to membranes once they come into contact with IRGM-depleted pathogen-containing vacuoles (29, 30). In IRGM-deficient mouse cells, cytoplasmic GKS proteins transition spontaneously into the GTP-bound state and form aggregates (30, 31). Aggregation depletes the pool of transportable GKS monomers, and IRGM-deficient mouse cells therefore fail to deliver GKS proteins, E3 ligases, and ubiquitin to *C. trachomatis* inclusions (25).

The *IRG* gene family consists of approximately 20 members in the mouse but has undergone a dramatic collapse in the primate lineage. The human genome encodes a single IRGM ortholog and lacks GKS-encoding genes entirely (27, 32). Because the murine IRG system is essential for the delivery of ubiquitin to *C. trachomatis* inclusions (25), the question arose whether human cells could ubiquitinate inclusions in spite of their diminished IRG system. We report here that human cells possess an IRG-independent system for the attachment of ubiquitin to inclusions. We further report that human cells can deliver ubiquitin to inclusions formed by the rodent-adapted pathogen *Chlamydia muridarum* but that the human-adapted species *C. trachomatis* is resistant to ubiquitination in human cells. These observations indicate that *C. trachomatis* has evolved strategies to interfere with the human-specific ubiquitination machinery.

## RESULTS

### *C. muridarum*, but not *C. trachomatis*, inclusions are frequently associated with ubiquitin in human epithelial cells.

**FIG 1:**
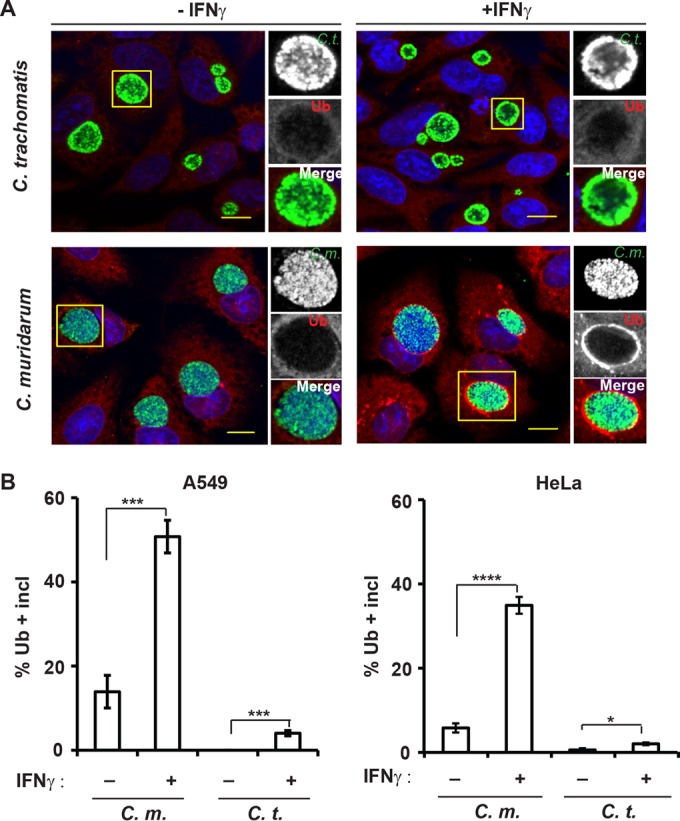
IFN-γ priming promotes ubiquitin deposition on inclusions in human cells. (A) A549 epithelial cells were infected with either *C. muridarum* (*C*.*m*.) or *C. trachomatis* (*C*.*t*.) and primed with IFN-γ (+IFNγ) (100 U/ml) at 3 hpi or left unprimed (−IFNγ). At 20 hpi, cells were stained for ubiquitin (Ub) with antibody FK2 (red), rabbit anti-Slc1 (green), and DNA (blue). Representative images of Ub-positive inclusions are shown. Bars = 10 μm. (B) Colocalization of ubiquitin (FK2) with inclusions (incl) in A549 cells and HeLa cells was quantified as described in Materials and Methods. At least 100 inclusions were counted for each condition. Data are representative of three independent experiments. Values are means ± standard deviations (SD) (error bars). Values that are statistically significantly different by two-tailed unpaired Student’s *t* test are indicated by a bar and asterisks as follows: *, *P* < 0.05; ***, *P* < 0.005; ****, *P* < 0.001.

Because IFN-γ-primed mouse cells coat *C. trachomatis* inclusions with a layer of ubiquitin (25), we asked whether human cells were similarly able to deposit ubiquitin on *C. trachomatis* inclusions. To answer this question, we infected two commonly used human epithelial cell lines, A549 and HeLa cells, with *C. trachomatis*. When cells were stained with the antiubiquitin antibody FK2 at 20 h postinfection (hpi), ubiquitin-positive *C. trachomatis* inclusions were not detected in naive A549 cells and extremely rare in naive HeLa cells (less than 1% ubiquitin-positive inclusions [Fig. 1A and B]). IFN-γ priming resulted in a moderate increase in the number of ubiquitin-positive *C. trachomatis* inclusions yet did not exceed 5% in either cell line (Fig. 1B). We then monitored ubiquitin staining of inclusions formed by the rodent pathogen *C. muridarum*, which is resistant to ubiquitination in IFN-γ-primed mouse cells (25). Unexpectedly, we detected ubiquitin-positive *C. muridarum* inclusions at relatively high frequencies (up to 15%) compared to *C. trachomatis* inclusions in naive A549 and HeLa cells. IFN-γ priming further increased the frequency of ubiquitin-positive *C. muridarum* inclusions to approximately 50% (Fig. 1A and B). These data demonstrated that IFN-γ priming in human cells—similar to mouse cells—prompts robust ubiquitination of inclusions. Yet, in clear contrast to mouse cells, human cells fail to efficiently coat *C. trachomatis* inclusions with ubiquitin. The latter observations could be explained if the pathways leading to inclusion ubiquitination differed between mice and humans and if *C. trachomatis* was specifically adapted to the human, but not the mouse, pathway.

### Ubiquitination of *C. muridarum* inclusions is independent of human IRGM.

**FIG 2:**
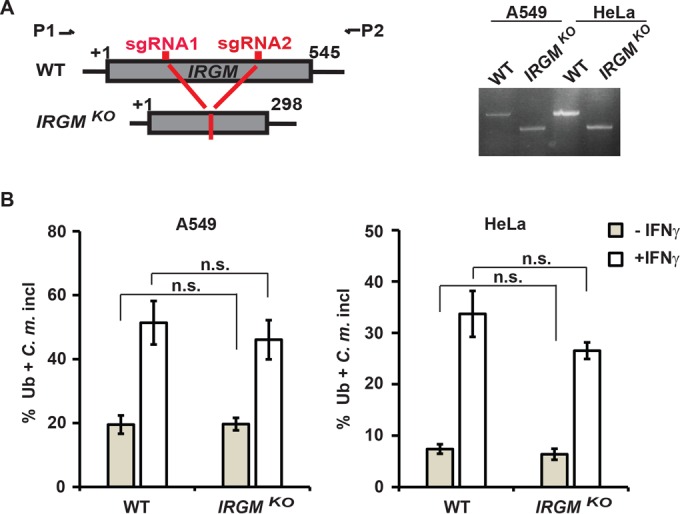
Human IRGM is dispensable for the ubiquitination of *C. muridarum* inclusions**.** (A) CRISPR/Cas9 genome editing was used to generate deletions of approximately 230 bp in the coding sequence of human *IRGM*. The locus was targeted using two small guide RNAs (sgRNA1 and sgRNA2), and individual cell clones were isolated from parental A549 and HeLa cell lines and confirmed for deletions in the *IRGM* gene by PCR. (B) Parental (wild type [WT]) and *IRGM*^KO^ (KO stands for knockout) A549 and HeLa cells were infected with *C. muridarum* (*C*. *m*.) and primed with IFN-γ (100 U/ml) at 3 hpi or left unprimed. At 20 hpi, the cells were stained for ubiquitin (FK2) and *C. muridarum* (anti-Slc1). Colocalization of ubiquitin with inclusions in A549 cells and HeLa cells was quantified as described in Materials and Methods. At least 100 inclusions were counted for each condition. Values that are not statistically significantly different (n.s.) by two-tailed unpaired Student’s *t* test are indicated. Data are representative of three independent experiments. Values are means ± standard deviations (SD) (error bars).

On the basis of the rationale outlined above, we asked whether the human and mouse pathways of inclusion ubiquitination were distinct. We showed previously that IRGM proteins were essential for the decoration of inclusions with ubiquitin and the killing of *C. trachomatis* in IFN-γ-primed mouse cells (25, 29). To determine whether the single human IRGM ortholog is required for the ubiquitination of inclusions, we generated A549 and HeLa clonal cell lines with large (~230-bp) deletions in the human *IRGM* coding region (Fig. 2A). The frequency at which *C. muridarum* inclusions stained positive for ubiquitin was not significantly altered in cell clones carrying deletions in the human *IRGM* gene (Fig. 2B), demonstrating that inclusion ubiquitination in human cells is IRGM independent and therefore mechanistically distinct from the murine pathway.

### Linear and branched ubiquitin, ubiquitin-binding proteins, and LC3 localize to *C. muridarum* inclusions in human cells.

**FIG 3:**
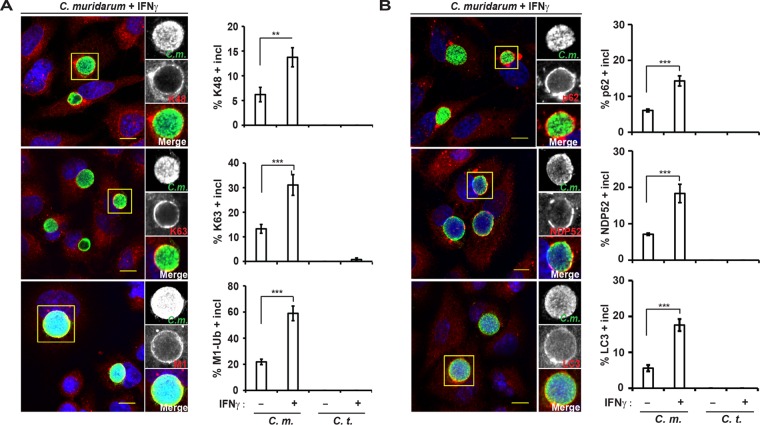
Distinct ubiquitin species, ubiquitin-binding proteins, and LC3 associate with *C. muridarum* inclusions in human cells. Wild-type A549 cells were infected with *C. muridarum* or *C. trachomatis* and primed with IFN-γ (100 U/ml) at 3 hpi or left unprimed. (A and B) At 20 hpi, cells were stained with the linkage-specific antiubiquitin antibodies against K48, K63, or linear ubiquitin (M1) (A) or with antibodies against the host defense proteins (p62) NDP52 or LC3 as well as anti-chlamydial LPS and DNA (Hoechst) (B). (Left) Representative images are shown. Bars = 10 μm. (Right) Colocalization of different host markers with inclusions in A549 cells was quantified. At least 100 inclusions were counted for each condition. Data are representative of three independent experiments. Values are means ± SD. Values that are significantly different by two-tailed unpaired Student’s *t* test are indicated by a bar and asterisks as follows: **, *P* < 0.01; ***, *P* < 0.005.

We previously reported that K48- and K63-linked polyubiquitin and the ubiquitin-binding protein p62 associate with inclusions in IFN-γ-primed mouse cells (25). In order to determine whether a similar ubiquitination pattern is observed in human cells, we stained *C. muridarum*-infected A549 cells with linkage-specific antibodies. We found that K48- and K63-linked ubiquitin accumulated at *C. muridarum*, but not *C. trachomatis*, inclusions and that this association was exacerbated by IFN-γ priming (Fig. 3A). These results thus confirmed the data obtained with FK2 (Fig. 1), an antibody that detects K48- and K63-linked, but not linear, ubiquitin (33, 34). Linear ubiquitin polymers form through conjugation between the C-terminal α-carboxyl group of an incoming ubiquitin and the α-amino group of the N-terminal methionine (M1) of substrate-bound ubiquitin (35). Staining with anti-M1 ubiquitin antibody revealed that a high percentage of *C. muridarum* inclusions are decorated with M1 (Fig. 3A). In addition to these distinct types of ubiquitin conjugates, we also detected the presence of the ubiquitin-binding proteins p62 and NDP52 as well as the ubiquitin-like protein LC3 at *C. muridarum*, but not *C. trachomatis*, inclusions (Fig. 3B). Similar to the ubiquitin-staining pattern, we found that the association of p62, NDP52, and LC3 with *C. muridarum* inclusions was enhanced in IFN-γ-primed cells, indicating a functional link between inclusion ubiquitination and the recruitment of these additional host proteins.

### Recruitment of host defense proteins p62, NDP52, and LC3 is restricted to ubiquitinated inclusions.

**FIG 4:**
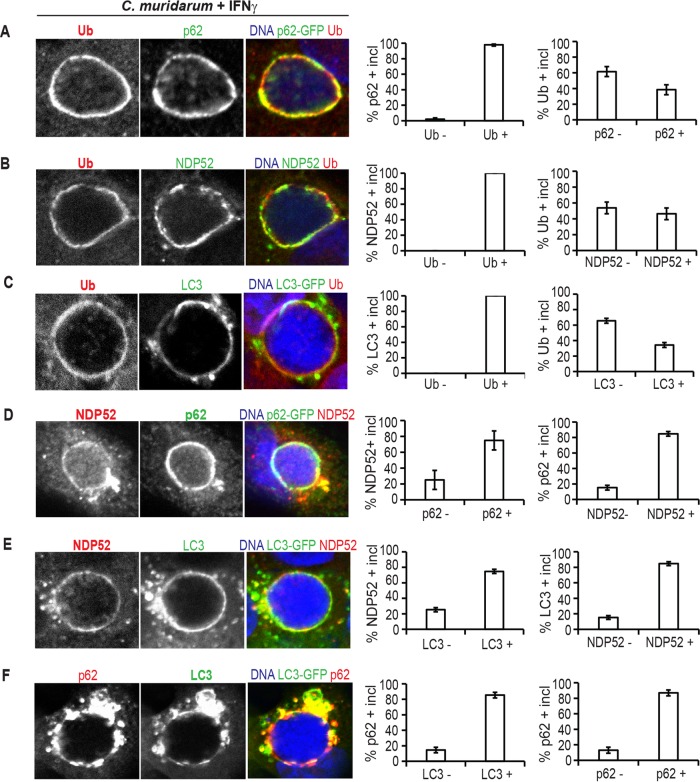
NDP52, p62, and LC3 colocalize with ubiquitin on *C*. *muridarum* inclusions in A549 cells. (A to F) A549 cells expressing p62-GFP (A and D) or LC3-GFP (C, E, and F) or untransfected cells (B) were infected with *C. muridarum*, primed with IFN-γ (100 U/ml) at 3 hpi, fixed at 20 hpi, and stained with antibodies against the indicated host proteins. Hoechst staining was used to visualize bacterial DNA of inclusions. Representative images of colocalization between indicated host markers at *C. muridarum* inclusions are shown. At least 50 inclusions were counted for each condition. Data are representative of three independent experiments. The values in the graphs are means ± SD (error bars). Ub −, ubiquitin negative; Ub +, ubiquitin positive.

To address the question of whether inclusion ubiquitination could be functionally linked to the recruitment of additional host defense proteins, we first assessed whether ubiquitin, ubiquitin-binding proteins, and LC3 colocalized at inclusions. We observed that p62, NDP52, and LC3 localized exclusively to ubiquitin-positive inclusions, while more than half of ubiquitin-positive inclusions stained negative for p62, NDP52, or LC3 (Fig. 4A to C). These data indicated that ubiquitination occurs upstream of the recruitment of p62, NDP52, and LC3. We further recorded that many (~70%), but not all, NDP52-decorated inclusions stained positive for p62 and vice versa (Fig. 4D). Similarly, most, but not all, NDP52- or p62-positive inclusions (70 to 80%) colocalized with LC3 (Fig. 4E and F). These data suggested that the recruitment of p62, NDP52, and LC3 to inclusions is largely independent of each other, resembling previous observations made for the association of p62 and NDP52 with *Salmonella*-containing vacuoles (36).

**FIG 5:**
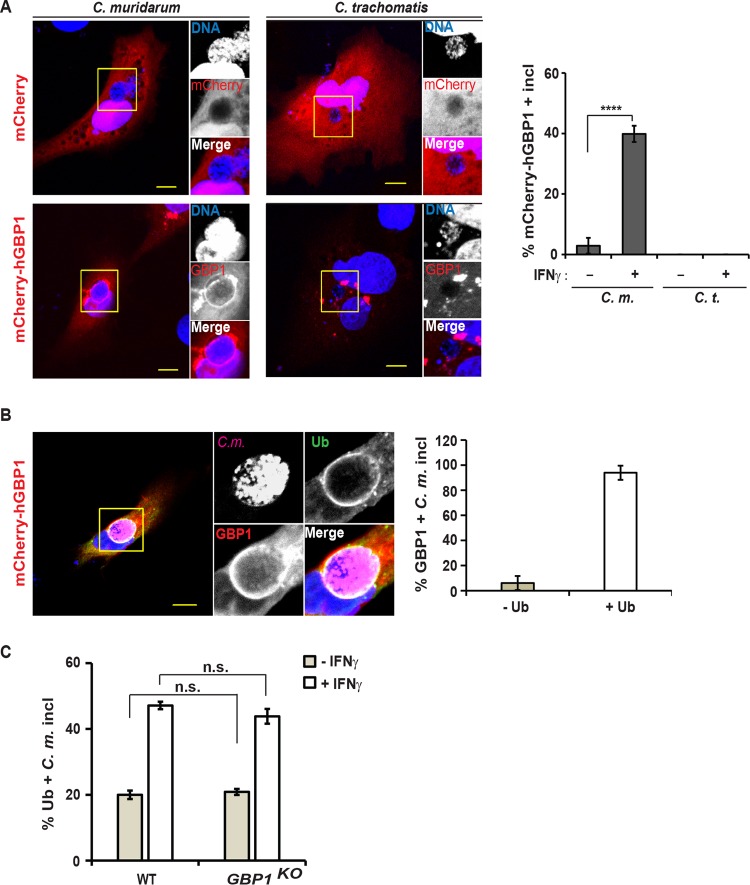
Human GBP1 targets ubiquitin-positive inclusions. (A) A549 cells were transfected with an mCherry-hGBP1 expression construct and 24 h later infected with *C. muridarum* or *C. trachomatis* at an MOI of 2 and primed with IFN-γ (100 U/ml) at 3 hpi or left unprimed. At 20 hpi, the cells were fixed and stained with Hoechst. (Left) Representative images are shown. Bars = 10 μm. (Right) Percentage of mCherry-GBP1-positive inclusions in mCherry-expressing cells was quantified. Data are representative of three independent experiments. Values are means ± SD (error bars) (*n* = 3). Values that are significantly different (*P* < 0.001) by Student’s *t* test are indicated by a bar and four asterisks. (B) Experiment was performed as described above for panel A, except for staining cells additionally with anti-ubiquitin (FK2) and anti-*Chlamydia* (Slc1) antibodies. (Left) Representative images are shown. (Right) Ubiquitin staining of hGBP1-positive inclusions was quantified. Data are representative of two independent experiments. Values are means ± SD (*n* = 3). (C) Parental (WT) and *GBP1*^KO^ A549 cells were infected with *C. muridarum* and primed with IFN-γ (100 U/ml) at 3 hpi or left unprimed. At 20 hpi, colocalization of ubiquitin (FK2) with inclusions was quantified. Data are representative of three independent experiments. Values are means ± SD. Values that are not significantly different (n.s.) are indicated.

We then examined whether members of the GBP family of host resistance factor colocalized with *Chlamydia* based on our previous finding that murine GBPs bind to ubiquitinated *C. trachomatis* inclusions in mouse cells (25). To assess whether human GBPs could similarly target ubiquitinated inclusions in human cells, we monitored the subcellular localization of human GBP1 by ectopically expressing mCherry-labeled human GBP1 (mCherry-hGBP1) fusion protein in A549 cells. We found mCherry-hGBP1 localized to *C. muridarum*, but not *C. trachomatis*, inclusions (Fig. 5A). Colocalization of mCherry-hGBP1 with *C. muridarum* increased from approximately 5% in naive cells to about 40% in IFN-γ-primed cells (Fig. 5A), mirroring the IFN-γ-induced increase in the number of ubiquitin-positive inclusions. Most hGBP1-positive *C. muridarum* inclusions (>90%) also stained with antiubiquitin antibody FK2 (Fig. 5B), suggesting that hGBP1 specifically targets ubiquitin-positive inclusions. As an alternative explanation, we considered the possibility that inclusion-resident hGBP1 itself was required for the attachment of ubiquitin to inclusions. Refuting this model, we found that the frequency at which inclusions were decorated with ubiquitin remained the same between wild-type and hGBP1-defiicent A549 cells (Fig. 5C), demonstrating that hGBP1 itself is not required for labeling inclusions with ubiquitin.

### Fusion with *C. trachomatis* inclusions reduces ubiquitination of *C. muridarum* inclusions.

**FIG 6:**
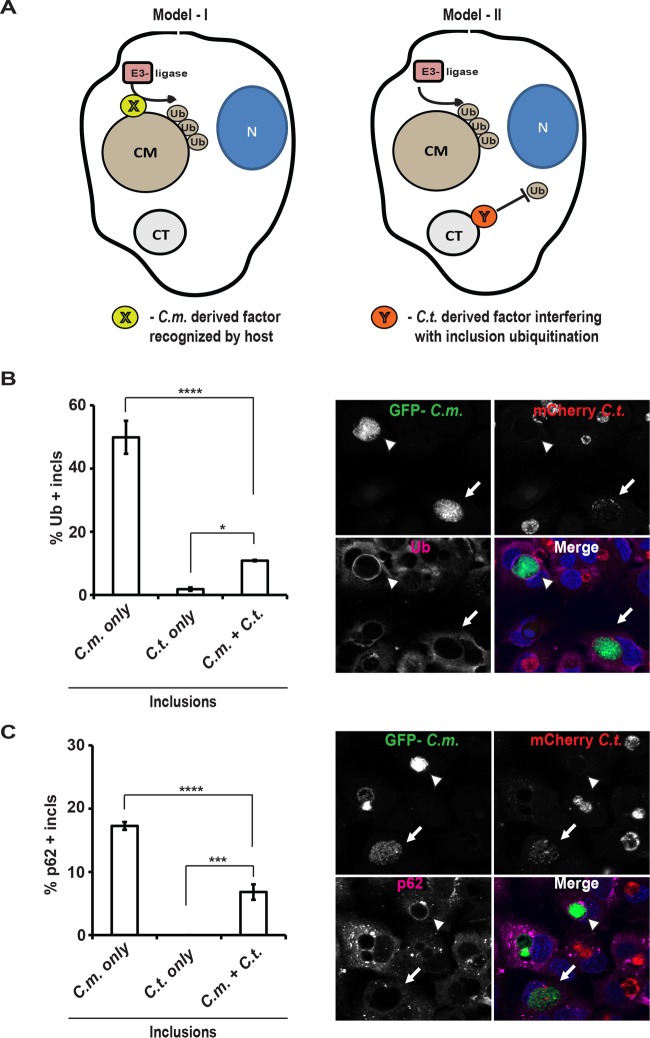
Fusion with *C. trachomatis* inclusions diminishes ubiquitination of *C. muridarum*-containing inclusions. (A) Two competing models to account for the preferential ubiquitin (Ub) targeting of *C. muridarum* (CM) versus *C. trachomatis* (CT) inclusions are depicted. N, nucleus. (B and C) A549 cells were coinfected with GFP^+^ *C. muridarum* (*C*.*m*.) (MOI of 3) and mCherry^+^ *C. trachomatis* (*C*.*t*.) (MOI of 5), primed with IFN-γ at 3 hpi, and fixed and stained with Hoechst and either antiubiquitin (FK2) (B) or anti-p62 (C) antibodies at 20 hpi. (Right) Representative images are shown. White arrowheads indicate ubiquitin- or p62-positive inclusions exclusively occupied by GFP^+^ *C. muridarum*. White arrows indicate inclusions containing a mixed population of GFP^+^ *C. muridarum* and mCherry^+^ *C. trachomatis*. (Left) For quantification, inclusions were categorized into three groups based on the type of bacteria they contained: GFP^+^ only (*C.m.* only), mCherry^+^ only (*C.t.* only), and GFP^+^ mCherry^+^ (*C.m.* + *C.t.*). Three independently infected wells were analyzed per data point and per well at least 30 inclusions of each category were scored for colocalization with ubiquitin or p62. Data are represented as means ± SD. Values that are significantly different by one-way ANOVA are indicated by a bar and asterisks as follows: *, *P* < 0.05; ***, *P* < 0.005; ****, *P* < 0.001.

Our data so far demonstrated that IFN-γ priming of human cells leads to the ubiquitination of *C. muridarum* inclusions and the subsequent recruitment of host defense proteins such as NDP52 and hGBP1. Two distinct models could explain why human host cells effectively ubiquitinate *C. muridarum*, but not *C. trachomatis*, inclusions. In the first model, a *C. muridarum*-derived factor X is directly recognized by the human host. This factor X would be predicted to be absent from *C. trachomatis* inclusions, which therefore remained largely devoid of ubiquitin. In the second model, both *C. muridarum* and *C. trachomatis* inclusions are bona fide targets of the host immune system, but only *C. trachomatis* is able to interfere with host-mediated ubiquitination through the activity of a *C. trachomatis*-derived factor Y (Fig. 6A). In order to differentiate between these two models, we took advantage of the ability of inclusions to fuse with each other in coinfected cells. To discern fused from unfused inclusions, we coinfected A549 cells with green fluorescent protein (GFP)-expressing *C. muridarum* and mCherry-expressing *C. trachomatis*. Next, we stained cells with antiubiquitin antibody and quantified the percentage of ubiquitin-positive inclusions containing GFP-positive (GFP^+^) or mCherry-positive (mCherry^+^) or a mixed population of both GFP^+^ and mCherry^+^ bacteria. We observed that the presence of *C. trachomatis* reduced the percentage of ubiquitin-positive *C. muridarum* inclusions from ~50% to ~10% (Fig. 6B) and also significantly reduced the percentage of p62-positive *C. muridarum* inclusions (Fig. 6C). While these data do not entirely dismiss the first model, they are more readily reconciled with the second model, in which a *C. trachomatis-*derived factor Y protects inclusions against host-mediated ubiquitination.

### IFN-γ priming activates a human host defense pathway that is effective against *C. muridarum*, but not *C. trachomatis*.

**FIG 7:**
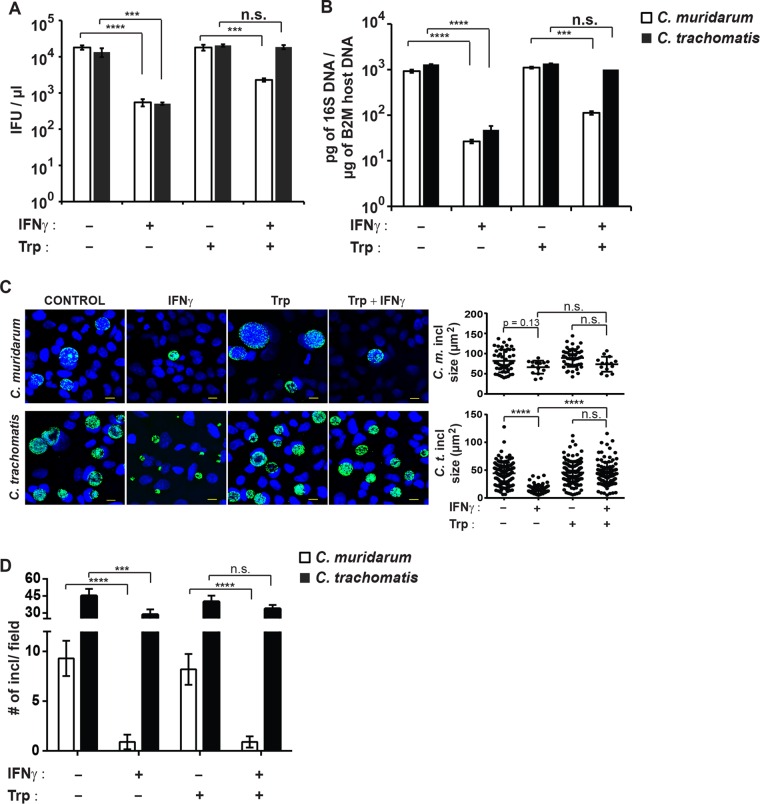
IFN-γ promotes the clearance of *C. muridarum* from human A549 cells in an IDO-independent manner. A549 cells were primed overnight with IFN-γ (100 U/ml) or left unprimed. Cells were infected with *C. muridarum* or *C. trachomatis* at an MOI of 1 in the presence (+) or absence (−) of excess tryptophan (Trp) (100 µg/ml). (A) The number of IFUs per microliter were measured from each culture condition at 27 hpi. (B) Bacterial burden was assessed by qPCR at 27 hpi. Values that were significantly different by two-tailed unpaired Student’s *t* test are indicated by a bar and asterisks as follows: ***, *P* < 0.005; ****, *P* < 0.001. Values that were not significantly different (n.s.) by two-tailed unpaired Student’s *t* test are also indicated. (C and D) Cells were infected as described above, fixed at 27 hpi, and stained with anti-LPS (green) and Hoechst (blue). ImageJ software was used to measure inclusion size (area in square micrometer in panel C) and inclusion numbers per randomly acquired image field (D). Representative images are shown in panel C (bars = 10 μm). Data are representative of two independent experiments. Values are means ± SD. Values that are significantly different by one-way ANOVA are indicated by a bar and asterisks as follows: ***, *P* < 0.005; ****, *P* < 0.001. Values that were not significantly different (n.s.) by one-way ANOVA are also indicated.

Our observations hitherto demonstrated that human epithelial cells mark *C. muridarum* inclusions with ubiquitin and a set of additional host defense proteins that include p62, LC3, and GBP1. We further showed that this process was significantly augmented by IFN-γ priming and that *C. trachomatis* can interfere with the recruitment of these antimicrobial host factors to inclusions. These observations thus led to the hypothesis that IFN-γ-primed human cells executed a cell-autonomous defense program that is effective against *C. muridarum* but ineffective against *C. trachomatis*. To test this hypothesis, we infected naive or IFN-γ-primed A549 cells with either *C. muridarum* or *C. trachomatis* and assessed bacterial burden by measuring infectious *Chlamydia* progeny (Fig. 7A) or by quantitative PCR (qPCR) quantification of *Chlamydia* DNA content (Fig. 7B). In agreement with previous reports (15, 16), we observed a greater than 1-log-unit reduction in chlamydial burden in IFN-γ-primed cells compared to naive cells (Fig. 7A and B). Because growth of *C. muridarum* and *C. trachomatis* was restricted equally in IFN-γ-primed A549 cells, we considered that the putative ubiquitin-dependent host defense pathway directed specifically against *C. muridarum* was masked by the IDO response.

IFN-γ priming of human epithelial cells induces the expression of IDO, an enzyme that depletes intracellular tryptophan stores and thereby restricts growth of the tryptophan auxotroph *Chlamydia*. Supplying excess tryptophan to the culture media is known to reverse the anti-*Chlamydia* effect of IDO (14–16). In agreement with these previous observations, we observed complete restoration of *C. trachomatis* burden in IFN-γ-primed A549 cells cultured in tryptophan-enriched media (Fig. 7A and B). However, tryptophan addition had only a limited effect on the number of inclusion-forming units (IFUs) generated by *C. muridarum* inside IFN-γ-primed A549 cells (Fig. 7A) and had no impact on *C. muridarum* burden as measured by qPCR (Fig. 7B). These data therefore demonstrated that IFN-γ-primed A549 cells repress growth of *C. muridarum*, but not *C. trachomatis*, independent of IDO-mediated tryptophan depletion.

We next asked whether this IDO-independent anti-*Chlamydia* defense pathway was bactericidal or bacteriostatic. IDO-mediated cell-autonomous immunity against *Chlamydia* acts in a bacteriostatic way, halting bacterial growth and thereby causing *C. trachomatis* inclusions to be smaller in size (Fig. 7C), while only minimally affecting the total number of inclusions under tryptophan-limiting culture conditions (Fig. 7D). In contrast to *C. trachomatis* infections, we found that the number of *C. muridarum* inclusions in IFN-γ-primed A549 cells was reduced by about 1 log unit, even under tryptophan-replete culture conditions (Fig. 7D). These data indicated that IFN-γ-primed A549 cells eliminated most *C. muridarum* inclusions and that the immune pathway described herein is independent of IDO and bactericidal in nature.

## DISCUSSION

The human pathogen *C. trachomatis* is the most common cause of sexually transmitted bacterial infections. No vaccine is currently available, and recurring, repeat, or chronic infections are frequent. The inefficacy of our immune system to clear *C. trachomatis* infections and its deficiency in establishing protective immune memory demonstrate that *C. trachomatis* can undermine human immunity (2–4, 8). However, how *C. trachomatis* escapes clearance by both innate and adaptive immune responses of its human host is poorly understood. Here, we describe IFN-γ-inducible ubiquitination of inclusions and the associated eradication of intracellular bacteria in human cells as a novel anti-*Chlamydia* host defense pathway and further demonstrate that *C. trachomatis* is resistant to this newly described human immune response.

IFN-γ-primed human epithelial cells express high levels of the tryptophan-degrading enzyme IDO. Tryptophan depletion restricts growth of tryptophan-auxotrophic *Chlamydia* species and induces *Chlamydia* to undergo dramatic physiological and morphological changes (11, 17, 37, 38). In response to tryptophan starvation, *C. trachomatis* upregulates a partial *trp* operon, which allows *C. trachomatis* to consume exogenous indole and thereby produce tryptophan needed for survival in a tryptophan-depleted environment (11, 18). The fine-tuned interaction between host IDO and the *C. trachomatis trp* operon is absent from the commonly used animal model, in which mice are infected with the *C. trachomatis*-related pathogen *C. muridarum*. *C. muridarum* lacks a *trp* operon, and mouse genital epithelial cells express little to no IDO (5, 7, 15, 22). The relationship between the *C. trachomatis trp* operon and the human IDO response thus beautifully illustrates how *C. trachomatis* is specifically adapted to its human host. Our current study shows that IFN-γ-primed human epithelial cells not only express IDO as part of their anti-*Chlamydia* defense program but also execute a second resistance pathway to which *C. trachomatis* evolved an immune evasion strategy.

**FIG 8:**
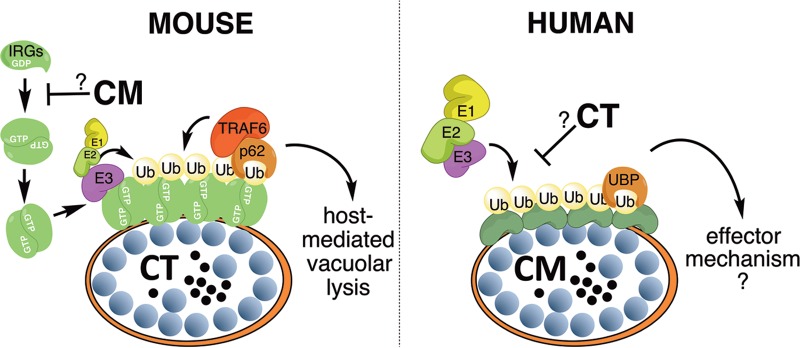
Distinct pathways in mice and humans control IFN-γ-inducible ubiquitination of inclusions. Inclusion ubiquitination in mouse cells is dependent on IFN-γ-inducible IRGs, which in their GTP-bound state form oligomers, bind to inclusions, and subsequently recruit E3 ubiquitin ligases such as TRAF6. By interfering with the recruitment of IRGs to its inclusion, the rodent pathogen *C. muridarum* (CM) blocks inclusion ubiquitination in mouse cells. Ubiquitinated inclusions undergo vacuolar lysis, leading to bacterial death (25). This current study demonstrates that IFN-γ priming also triggers inclusion ubiquitination in human cells, albeit by an IRG-independent mechanism. Whereas *C. muridarum* is susceptible to this IRG-independent pathway, the human pathogen *C. trachomatis* (CT) is resistant. Inclusion ubiquitination in human cells correlates with the elimination of inclusions from infected cells, but the underlying cellular mechanism is unknown. Ub, ubiquitin; UBP, ubiquitin-binding proteins; E1, ubiquitin-activating enzyme; E2, ubiquitin-conjugating enzyme.

While IDO induction robustly restricts chlamydial growth, previous examinations of an array of human cell lines suggested that additional, uncharacterized IFN-γ-inducible anti-*Chlamydia* pathways may exist (15). Our study reports that IFN-γ priming triggers the deposition of ubiquitin on inclusions in human cells. The coating of intracellular pathogens with ubiquitin is a conserved defense mechanism found in host organisms as diverse as fruit flies and humans (39, 40). We recently demonstrated that IFN-γ priming of mouse cells leads to the ubiquitination of *C. trachomatis* inclusions (25). While the ubiquitination of *C. trachomatis* inclusions in mouse cells requires murine IRGM1 and IRGM3 proteins (25), we demonstrate here that ubiquitination of *C. muridarum* inclusions in human cells is independent of human IRGM (Fig. 2). These results reveal that mouse and human cells evolved separate mechanisms to encapsulate inclusions within a layer of ubiquitin (Fig. 8). Because the host machinery for inclusion ubiquitination is different in mice and humans, the corresponding microbial evasion mechanisms must be adapted to the host species. This argument is supported by our findings. We previously demonstrated that *C. muridarum* evades cell-autonomous immunity in mice through the inactivation of the murine IRG system (25, 28), a strategy that is ineffective in human cells. *C. trachomatis* on the other hand fails to inactivate the murine IRG systems, rendering it susceptible to ubiquitination in mouse cells (25), but it has the ability to escape from the IRG-independent ubiquitination response of human cells (Fig. 8). To understand how different *Chlamydia* species escape from inclusion ubiquitination in their respective hosts, we will need to identify the chlamydial factors that enable evasion of inclusion ubiquitination. Forward genetic screens using recently developed *Chlamydia* mutant libraries (41–43) provide one possible avenue to achieve this goal.

A second area of future research will be focused on the identification and characterization of human factors that mediate inclusion ubiquitination and the cellular events that follow. Recent reports demonstrated that IFN-γ priming of human cells promote the ubiquitination of parasitophorous vacuoles (PVs) formed by the protozoan pathogen *Toxoplasma gondii* (44, 45), albeit by an unknown mechanism. IFN-γ-inducible cell-autonomous immune responses directed against *Chlamydia* and *Toxoplasma* have so far been found to be remarkably similar, as both types of pathogens are susceptible to the murine IRG and human IDO responses (46). On the basis of these precedents, it is tempting to speculate that the same molecular machinery drives the ubiquitination of both *Toxoplasma* PVs and inclusions in human cells, a hypothesis that will await future testing. Similar to our findings with *C. muridarum*, Selleck and colleagues showed that ubiquitinated *Toxoplasma* PVs corecruit ubiquitin-binding proteins and the autophagy marker LC3 (45). The same study demonstrated that parasites inside ubiquitin-positive PVs are moderately impeded for growth. However, in agreement with other work (47, 48), the IDO-independent effects of IFN-γ priming on overall parasitic burden remain negligible. In contrast to the moderate effects on *Toxoplasma* burden, we find the IFN-γ-inducible, IDO-independent anti-*Chlamydia* response to reduce bacterial burden by approximately 10-fold. This dramatic reduction in burden correlates with a 10-fold decrease in the number of inclusions, suggesting that IFN-γ-primed human cells destroy and remove inclusions, similar to what was observed in mouse cells (25). The precise mechanism by which the human host eliminates inclusions is currently unknown. Similar to the murine defense mechanism (25), inclusions inside IFN-γ-primed human epithelial cells may lyse and thereby release bacteria into the host cell cytosol for xenophagic destruction. Alternatively, inclusions may undergo acidification, as reported previously for inclusions formed inside IFN-γ-primed human macrophages (49). The study of Al-Zeer et al. (49) reported that hGBP1 and hGBP2 were required for *C. trachomatis* inclusion acidification in human macrophages, suggesting that *C. trachomatis* fails to counteract cell-autonomous host defense in human macrophages as it does in human epithelial cells. Future studies will have to determine whether inclusion acidification constitutes an effector pathway downstream of inclusion ubiquitination, or alternatively, a ubiquitination-independent pathway unique to macrophages.

In summary, our study describes a novel cell-autonomous immune response targeting *Chlamydia* inside human epithelial cells. The activation of this ubiquitin-based immune response is distinct from a similar ubiquitination pathway that we previously described in mouse cells (25). Therefore, mice and humans appear to have evolved distinct but functionally convergent systems to battle infections with intracellular *Chlamydia*. We further demonstrate that *C. trachomatis* is resistant to inclusion ubiquitination in human epithelial cells. As we will define mechanisms by which *C. trachomatis* can block the human-specific immune responses, it is hoped that our research will pave the way toward novel treatment options and improved vaccine strategies to minimize the impact of *C. trachomatis* infections on human health.

## MATERIALS AND METHODS

### *Chlamydia* strains, bacterial plasmids, and transformation.

*C. trachomatis* LGV-L2 and *C. muridarum* Nigg were propagated in Vero cells as described previously (29). *C. trachomatis* LGV-L2 was transformed with plasmid pGFP::SW2 or p2TK2-SW2-mCherry, and *C. muridarum* Nigg was transformed with plasmid pGFP::CM (50–52). Transformations were performed essentially as described previously (50). Briefly, purified elementary bodies (EBs) and plasmid were mixed in 200 µl CaCl_2_ buffer (10 mM Tris [pH 7.4] and 50 mM CaCl_2_) and incubated for 30 min at room temperature. One hundred microliters of this mixture was then added to a single well in a six-well plate together with 2 ml of Dulbecco’s modified Eagle’s medium (DMEM) plus 10% fetal calf serum (FCS). At 12 h postinfection (hpi), the medium was replaced with medium containing 1 µg/ml ampicillin. Passage 0 infections were harvested at 30 hpi and immediately used to infect a new six-well plate of McCoy cells. This process was repeated two more times, and at passage 4, fluorescent *Chlamydia* organisms were observed, plaque cloned twice, and expanded for future experiments. The presence of plasmid was confirmed by PCR.

### Host cell culture, *Chlamydia* infections, and measurements of bacterial burden.

HeLa and A549 cells were cultured in Dulbecco’s modified Eagle’s medium supplemented with 5% heat-inactivated fetal bovine serum (FBS). Infections with *C. trachomatis* and *C. muridarum* were performed at a nominal multiplicity of infection (MOI) of ~2 followed by treatment with 100 U/ml of IFN-γ at 3 hpi or as indicated, essentially as described previously (29). For coinfection experiments, A549 cells were infected with mCherry-expressing *C. trachomatis* and GFP*-*expressing *C. muridarum* at a MOI ratio of 5:3, followed by treatment with 100 U/ml of IFN-γ at 3 hpi. Burden was assessed by qPCR or inclusion-forming unit (IFU) assays. To assess chlamydial DNA content, total nucleic acid was prepared from trypsinized cell pellets using the QIAamp DNA minikit from Qiagen (Valencia, CA, USA). Samples were then subjected to SYBR green qPCR on an ABI 7000 sequence detection system to assess the amount of 16S *Chlamydia* and B2M host DNA in the sample. *Chlamydia* 16S DNA was detected through use of the following primers as described previously (20): 16S forward primer (5′ GGA GGC TGC AGT CGA GAA TCT 3′) and 16S reverse primer (5′ TTA CAA CCC TAG AGC CTT CAT CAC A 3′). Human B2M DNA was detected using the following primer sequences as described previously (53): B2M forward primer (5′ TGC TGT CTC CAT GTT TGA TGT ATC T 3′) and B2M reverse primer (5′ TCT CTG CTC CCC ACC TCT AAG T 3′). Standard curves were generated in parallel from known amounts of *C. trachomatis* and human DNA, and these curves were used to calculate the mass (in picograms) of *Chlamydia* DNA per unit mass (in micrograms) of human DNA per sample. IFU assays were performed essentially as described previously (20). Briefly, wild-type (WT) A549 and HeLa cells were primed or left unprimed overnight and infected with *C. muridarum* or *C. trachomatis* in 24-well plates. At 27 hpi, infected monolayers were lysed with water and physical dislodgement and suspended into 0.25 ml SPG buffer (220 mM sucrose, 12.5 mM phosphate, 4 mM l-glutamic acid, pH 7.5). Suspensions were transferred to sterile Eppendorf tubes and then sonicated. Samples were stored at −80°C. For quantification, samples were serially diluted, and dilutions were used to infect confluent monolayers of Vero cells in triplicate. The next day, samples were fixed with methanol and stained with mouse monoclonal anti-*Chlamydia* lipopolysaccharide (LPS), and host cell nuclei were labeled with Hoechst stain. Inclusions were visualized by using a microscope and counted in 10 fields per well for the calculation of infectious titer.

### Immunocytochemistry.

Immunocytochemistry was performed essentially as described previously (29). The cells were washed three times with phosphate-buffered saline (PBS) (pH 7.4) prior to fixation. The cells were fixed either with methanol or with 4% paraformaldehyde (PFA) (wt/vol) for 20 min at room temperature (RT). In all experiments involving PFA fixation, fixed cells were permeabilized/blocked with 0.05% (wt/vol) saponin and 2% (wt/vol) bovine serum albumin (BSA) in PBS (SBP) for 30 min at RT. The cells were stained with the indicated primary antibodies: rabbit polyclonal anti-p62/SQSTM1 (PMO45; MBL International) at 1:500, mouse monoclonal anti-p62/SQSTM1 (Abnova) at 1:500, mouse antiubiquitin (FK2; ENZO) at 1:50, rabbit monoclonal antiubiquitin K48 (Millipore) at 1:500, rabbit monoclonal anti-K63 (Millipore) at 1:100, rabbit polyclonal anti-NDP52 (Calcoco2; Abnova) at 1:200, mouse monoclonal anti-chlamydial LPS (catalog no. 1681; Santa Cruz) at 1:50, rabbit anti-Slc1 (Ct043) at 1:100, rabbit monoclonal anti-linear ubiquitin clone 1E3 (Millipore) at 1:50, and rabbit polyclonal anti-LC3 (PMO36; MBL International) at 1:500. The cells were then stained with Alexa Fluor-conjugated secondary antibodies (Molecular Probes/Invitrogen). Nucleic DNA and bacterial DNA were stained with Hoechst 33258 according to the manufacturer’s protocol. Stained cells were washed with PBS, mounted on microscope slides with Mowiol (Sigma), and allowed to cure overnight. To monitor the subcellular localization of human GBP1, cells were transfected with a previously described mCherry-hGBP1 expression construct (54). Cells were imaged using either a Zeiss LSM 510 inverted confocal microscope or a Zeiss Axioskop 2 upright epifluorescence microscope. Colocalization of proteins with inclusions was quantified in at least three independent experiments. In each experiment, at least 10 randomly selected fields were imaged for each experimental condition and cell type. To determine the frequency with which ubiquitin (FK2, M1, K48, and K63), p62, NDP52, and LC3 proteins colocalize with inclusions, at least 100 inclusions were assessed for each experimental condition and cell type for every biological replicate. The fraction of ubiquitin-, p62-, NDP52- or GBP1-positive vacuoles was determined for each field by dividing the number of labeled vacuoles by the total number of vacuoles. To determine the number of inclusions per field, eight randomly selected microscopic fields were counted per condition. To determine inclusion size, inclusions in eight randomly selected fields were examined per condition, and area in square micrometers was calculated using ImageJ software.

### CRISPR gene deletion cell lines.

In order to make *IRGM* loss-of-function mutations, we utilized clustered regularly interspaced short palindromic repeat (CRISPR)/Cas9 technology with a pair of guide RNAs (gRNAs) that target *IRGM* near the 5′ and 3′ ends of the IRGM open reading frame (ORF). gRNAs were cloned into the human codon-optimized *Streptococcus pyogenes* (hSpCas9)-encoding vector pX330 (55), using BbsI restriction endonuclease in conjunction with preannealed or T4 polynucleotide kinase phosphorylated oligonucleotides containing compatible ends. The resulting constructs, pX330-*IRGM* guide 1 (target, 5′-ATCAGTGCCCTTCGAAACAC-3′) and pX330-*IRGM* guide 2 (target, 5′-GATGCTTGCCAAAACCGCTG-3′) were predicted to anneal at *IRGM* at positions +148 to 167 and positions +384 to 403, respectively. These constructs were cotransfected into A549 and HeLa cells using Lipofectamine LTX to introduce deletions of approximately 230 bp within the *IRGM* coding region. This deleted region encompasses a large portion of the predicted IRGM GTPase domain, including the G2 box/Switch I region and the G3 box/Switch II region (27, 32). Individual clones were isolated from the heterogeneous transfected population through serial dilution in 96-well plates. Wells containing single colonies (i.e., isolated clones) were identified by light microscopy. Genomic DNA was isolated from clonal populations using a DNeasy blood and tissue kit (Qiagen), and clones were screened for *IRGM* deletions through PCR with primers that flank the *IRGM* open reading frame. The screening primers used are listed as IRGM-Mutant-Screen-F (F stands for forward) (5′-GCCTCAGCCTCCTGTATTAGCTGG-3′) and IRGM-Mutant-Screen-R (R stands for reverse) (5′-GACAGGAATTAGTATTCACATAC-3′). A *GBP1*-deficient A549 cell line was previously reported (54).

### Statistical analysis.

Where designated, statistical significance was determined using the unpaired Student’s *t* test or two-way analysis of variance (ANOVA), as appropriate. Values of *P* < 0.05 were considered significant. Results are represented as means ± standard deviations (SD).
